# Selective logging: does the imprint remain on tree structure and composition after 45 years?

**DOI:** 10.1093/conphys/cov012

**Published:** 2015-03-24

**Authors:** Oyomoare L. Osazuwa-Peters, Colin A. Chapman, Amy E. Zanne

**Affiliations:** 1 Department of Biology, One University Boulevard, University of Missouri Saint Louis, Saint Louis, MO 63121, USA; 2 Department of Anthropology and School of Environment, McGill University, Montreal, Quebec, Canada H3A 2T7; 3 Wildlife Conservation Society, 2300 Southern Boulevard, Bronx, NY 10460, USA; 4 Department of Biological Sciences, The George Washington University, 2023 G Street NW, Washington, DC 20052, USA; 5 Center for Conservation and Sustainable Development, Missouri Botanical Garden, PO Box 299, Saint Louis, MO 63166, USA

**Keywords:** Functional traits, historical logging event, Kibale National Park, light intensity, wood density

## Abstract

Selective logging of tropical forests is increasing in extent and intensity. The duration over which impacts of selective logging persist, however, remains an unresolved question, particularly for African forests. Here, we investigate the extent to which a past selective logging event continues to leave its imprint on different components of an East African forest 45 years later. We inventoried 2358 stems ≥10 cm in diameter in 26 plots (200 m × 10 m) within a 5.2 ha area in Kibale National Park, Uganda, in logged and unlogged forest. In these surveys, we characterized the forest light environment, taxonomic composition, functional trait composition using three traits (wood density, maximum height and maximum diameter) and forest structure based on three measures (stem density, total basal area and total above-ground biomass). In comparison to unlogged forests, selectively logged forest plots in Kibale National Park on average had higher light levels, different structure characterized by lower stem density, lower total basal area and lower above-ground biomass, and a distinct taxonomic composition driven primarily by changes in the relative abundance of species. Conversely, selectively logged forest plots were like unlogged plots in functional composition, having similar community-weighted mean values for wood density, maximum height and maximum diameter. This similarity in functional composition irrespective of logging history may be due to functional recovery of logged forest or background changes in functional attributes of unlogged forest. Despite the passage of 45 years, the legacy of selective logging on the tree community in Kibale National Park is still evident, as indicated by distinct taxonomic and structural composition and reduced carbon storage in logged forest compared with unlogged forest. The effects of selective logging are exerted via influences on tree demography rather than functional trait composition.

## Abstract (precis)

We investigated the extent to which a selective logging event continues to leave its imprint on different components of an East African forest 45 years later. Selectively logged forests had higher light, fewer stems, less biomass, and different species abundance, but did not differ from unlogged forest in species traits (maximum height and diameter, and wood density). The persistent effects of selective logging in this forest were exerted via influences on number and size of stems rather than species traits.

## Introduction

Selective logging, the targeted harvesting of commercially valuable timber species in a single cutting cycle, is an increasingly important component of the human footprint on tropical forests; its extent and intensity are on the increase (Asner *et al.*, 2009; Gibson *et al.*, 2011). According to recent estimates, at least 390 000 000 ha of tropical humid forests were selectively logged as of 2009 (Asner *et al.*, 2009), an area slightly larger than the size of India. These estimates are likely to be conservative because clandestine selective logging operations are extensive and largely undocumented. For example, more than half of the timber harvested from five major timber-producing countries (Brazil, Cameroon, Ghana, Indonesia and Malaysia) was illegally extracted in 2009 (Rands *et al.*, 2010). Selective logging is probably expanding as a result of rising global demands for timber products, providing large revenues for developing economies (Gibson *et al.*, 2011; Putz *et al.*, 2012).

The current prevalence and potential for continued expansion of selective logging has led to a call to understand its impacts on tropical forests better, particularly in terms of biodiversity conservation and carbon sequestration (Picard *et al.*, 2012). The authors of several recent studies argue that its impacts are relatively benign in comparison to other uses of forests and selectively logged tropical forests have a high conservation value second only to pristine tropical forests (Gibson *et al.*, 2011; Putz *et al.*, 2012; Ramage *et al.*, 2013). Nevertheless, selective logging has known immediate impacts on the taxonomic, structural and functional aspects of tropical forests. Taxonomically, selective logging can shift the composition and relative abundance of species (Baraloto *et al.*, 2012), including a reduction in species richness (Clark and Covey, 2012) and a shift in the dominance of particular lineages (Berry *et al.*, 2010; Okuda *et al.*, 2003). Structural effects of selective logging include homogenization of canopy structure (Okuda *et al*., 2003), reduction of stem density (Cannon *et al.*, 1998; Slik *et al.*, 2002; Hall *et al.*, 2003) and total basal area (Bonnell *et al.*, 2011) and loss of large trees, with a shift towards medium-sized trees (Okuda *et al*., 2003; Bonnell *et al.*, 2011), consequently reducing above-ground biomass (AGB; Lasco *et al.*, 2006; Blanc *et al.*, 2009; Berry *et al.*, 2010; Lindner and Sattler, 2012). The effect of selective logging on the capacity of tropical forests to maintain ecosystem function has been examined through characterization of shifts in dominance of predefined functional groups centred on plant attributes such as shade tolerance (Hall *et al.*, 2003), successional status (Bonnell *et al.*, 2011) or wood strength (Verburg and van Eijk-Bos, 2003). A handful of studies have used a more explicit continuous trait-based approach to demonstrate that tree communities respond to disturbance from selective logging by shifts in the range of functional trait values found in the community (Mayfield *et al.*, 2010; Baraloto *et al.*, 2012; Carreño-Rocabado *et al.*, 2012; Mouillot *et al.*, 2013).

Less clear than the immediate impacts of selective logging on tropical forests is the duration over which these impacts persist, given that trees are long lived and most studies are conducted within the first two decades after logging (Gibson *et al.*, 2011; Kormos and Zimmerman, 2014). Gibson *et al.* (2011) also highlight a regional bias in the tropical land-use change literature, most of which focuses on Southeast Asian and Neotropical forests, with few studies in Africa. Yet, African forests differ from other tropical forests in several ways, including the presence of older soils (Richards, 1996), a smaller regional species pool (Richards, 1996; Chapman *et al.*, 1999) and historically fewer and smaller disturbances (Chapman *et al.*, 1999). The degree of logging damage in Africa is relatively lower than that in Southeast Asia and higher than in the Neotropics (Picard *et al.*, 2012). To develop a comprehensive understanding of the long-term impacts of selective logging on the conservation value of tropical forests, we need more empirical studies exploring long-term effects on selectively logged African forests.

Here, we investigate the extent to which impacts of selective logging performed 45 years ago persist in an East African forest. We hypothesize that recovery from disturbance will be slow, such that imprints of selective logging will still be evident on the taxonomic, structural and functional components after almost half a century. We consider effects on understory light availability and community structural, taxonomic and functional composition. Understory light is strongly influenced by forest canopy structure, and typically differs between old-growth and disturbed forests because their canopies are open to varying extents (Nicotra *et al.*, 1999). These different light conditions tend to favour different combinations of functional traits or ecological strategies, such that disturbed forests may be dominated by species with resource-acquisitive or disturbance-adapted strategies, leading additionally to taxonomic and structural differences. To capture such differences in functional composition between species in logged and unlogged plots, we focused on three traits. Wood density (WD; in grams per cubic centimetre), a measure of a tree's dry carbon investment per unit volume, is a key indicator of the wood economic spectrum owing to its strong connection with several aspects of a plant's ecology, including growth rate, carbon allocation, structural stability, hydraulic conductivity and disease or pest resistance (Chave *et al*., 2009). The other two traits, plant maximum height (*H*_max_; in metres) and maximum diameter at breast height (DBH_max_; in centimetres), are crucial components of a species' light-competitive ability and carbon-gain strategy (King *et al.*, 2006; Wright *et al.*, 2007; Moles *et al.*, 2009). Both WD and adult stature vary with species' light requirements and along a successional continuum (Falster and Westoby, 2005; Chave *et al.*, 2009).

We predict that a logged forest will have the following characteristics: (I) more open canopy and higher light levels; (II) greater stem density, but less total basal area and above-ground biomass because higher light conditions will favour recruitment of more stems per unit area and unlogged forest will have more large trees; (III) distinct tree size distribution, with a relatively high frequency of mid-sized trees in comparison to the typical ‘reverse J-shaped’ tree size distributions for old-growth unlogged forest (Denslow, 1995); and (IV) distinct taxonomic and functional composition because it is at an earlier stage of succession and dominated by species with a resource-acquisitive strategy, whereas unlogged forests will be dominated by species with a resource-conservative strategy. Prediction IV implies that logged forests will have lower community-weighted mean values for WD, *H*_max_ and DBH_max_ compared with unlogged forest.

## Materials and methods

### Study site

This study was conducted in Kibale National Park (KNP; 795 km^2^) in south-western Uganda (Chapman *et al.*, 2010a). It is composed predominantly of mature moist semi-deciduous and evergreen forest, but includes a variety of other habitats, such as grassland, woodland, lakes and wetlands, colonizing forest and regrowth in areas previously planted with exotic trees (Chapman and Lambert, 2000). Kibale National Park receives an average of 1643 mm rainfall annually (1990–2013; C. A. Chapman and L. J. Chapman, unpublished data collected at Makerere University Biological Field Station), with two rainy seasons from March to May and September to November (Chapman *et al.*, 2010a). Temperature ranges between a mean daily minimum of 15.5°C and maximum of 23.7°C. Kibale National Park is divided into compartments, which were subjected to varying degrees of logging and have experienced different restoration efforts (Struhsaker, 1997; Chapman *et al.*, 2010a). Our study involved three compartments within KNP, as follows: (i) K-30 (282 ha) is relatively disturbance free in recorded history, at least from humans, and is typically considered a mature old-growth forest; (ii) K-14 (405 ha) was selectively logged between May and December 1969, but in a spatially heterogeneous manner, so that some areas (Mikana) experienced heavy logging, with the removal and damage of up to 25% of all trees, while other areas were largely untouched (lightly logged areas); and (iii) K-15 (347 ha) experienced high-intensity selective logging between September 1968 and April 1969, resulting in removal and damage of up to 50% of all trees. All of these compartments have been classified as *Parinari* forest (Osmaston, 1959), are located closely together (within 1500 m), and prior to logging exhibited high levels of structural similarity in cumulative basal area, canopy cover and stem density (Kingston, 1967; Bonnell *et al.*, 2011).

### Vegetation plots

Twenty-six permanent vegetation plots were randomly established within the existing trail system in KNP in December 1989. Each plot was 200 m × 10 m, with a trail running down the middle of its length. The locations of the 26 plots were unevenly distributed across the three compartments; 11 plots were located in K-30, six were in the lightly logged areas and four in the heavily logged (Mikana) parts of K-14, and five were in K-15. In this study, we assigned the 17 plots in K-30 and the lightly logged areas of K-14 to the unlogged plot category, while the nine plots in the Mikana part of K-14 and in K-15 were assigned to the logged category. The addition of plots in the lightly logged areas of K-14 to the unlogged plot category is informed by earlier works that established that the lightly logged forest suffered little damage from the logging event based on stump and gap enumeration (Kasenene, 1987; Chapman and Chapman, 1997; Bonnell *et al.*, 2011).

### Data collection

#### Forest light-intensity conditions

For 10 focal plots (five randomly selected from the 17 unlogged and five randomly selected from the nine logged plots), light was measured from June to August 2011 between 09.00 and 14.00 h. Light intensity was measured on both sides of each plot, extending from the dividing trail in the middle. Measurements were made at 10 m intervals underneath the forest canopy at 2 m above ground level as photosynthetically active radiation using an LI250 light meter and an LI-190SA quantum sensor (Licor, Lincoln, NE, USA). This photosynthetically active radiation was expressed relative to open light conditions, which were concurrently measured in the open at the Makerere University Biological Field Station using an HOBO light-intensity data logger. This resulted in 42 light-intensity measurements per plot.

#### Forest composition and structure

All 26 plots were surveyed between March and May 2013. Surveys of each plot involved recording the DBH for all tree stems with DBH ≥10 cm (Chapman *et al.*, 2010a). Trees were identified using recognized taxonomic keys (Polhill, 1952; Hamilton, 1991; Katende *et al.*, 1995; Lwanga, 1996), and species names were updated using The Plant List (http://www.theplantlist.org/). Voucher specimens for all trees were given to Makerere University Biological Field Station, and new specimens are currently being collected for the new herbarium at the Field Station. The resulting data set contains information on stem number, species composition and species relative abundances for each plot.

#### Species' functional traits

For the same 10 plots used for forest light-intensity conditions, we measured WD and height for all species between June and August 2011. While many species found in the 10 focal plots were also found in the remaining 16 plots, this trait data set excluded rare species, which although present in one or more of the 26 vegetation plots did not occur in the focal plots. Field-measured WD was available for only 60 species, height data for 58 species and DBH for all 86 species in the 2013 census.

##### Diameter at breast height

Tree trunk diameter was measured at 1.3 m above ground level for all stems ≥10 cm in the 26 plots during the 2013 census of vegetation plots. Species DBH_max_ was the largest DBH value recorded of all individuals of a species in these 26 plots.

##### Wood density

Wood samples were extracted at 1.3 m above ground level with a 30.5  cm increment borer from up to five (or fewer when not available) upright adult individuals per species within each of the 10 focal plots (for a total of 687 trees). Extracted wood cores ranged from 4 to 8 cm in length, and each core was broken into 2 cm segments, with the number of segments dependent on the length of the core. For each segment, fresh volume was determined using the water-displacement method, and dry mass was determined after oven drying to constant mass at 105°C (Osazuwa-Peters *et al.*, 2011). Wood density for each segment was computed as dry mass divided by fresh volume, and averaged over all segments that made up a core to determine a mean WD per tree. Species mean WD was then calculated as the average WD value of all individuals of a species pooled across the focal 10 plots. To obtain estimates for the remaining 26 unmeasured species found in the 16 non-focal plots, we obtained species-, genus- or family-level (depending on availability) WD averages from the Global Wood Density database subset to African region only (Zanne *et al.*, 2009). This African-region subset of the Global Wood Density Database contained WD values for six of the missing species, as well as genus-level WD values for nine species and family-level WD values for the remaining 11 species.

##### Tree height

Height was measured as the distance between the base and top of a tree for at least five (or fewer when unavailable) healthy adult individuals per species within each focal plot using a vertex hypsometer (Vertex IV, Haglöf, Sweden). Height values were obtained for 892 trees in the 10 plots. The maximum height of a species was then determined as the greatest height recorded across all individuals of a species (King *et al.*, 2006) from the pool of the 10 plots. Measurements of DBH were available for 622 of the 892 stems with height data. Height was regressed on DBH for this subset to obtain a forest-wide regression that was then used to interpolate height for species with DBH but missing height values. The regression (*r*^2^ = 0.31, *P* < 0.01) relationship was as follows:

(1) $$\hat{H_{\max}}=9.753326+(0.052898\times {\text{DBH}}_{\max})$$

This relationship was used to predict *H*_max_ from species DBH_max_ for the 28 species with missing maximum height data.

### Data analysis

Any variables demonstrating log-normal distributions were natural logarithmically transformed (see section on Structural composition).

#### Light environment

The 42 light-intensity measures per plot were averaged to give a mean estimate of light conditions underneath the forest canopy within each plot. We used a Student's unpaired *t*-test to compare mean light intensity between logged and unlogged plots.

#### Taxonomic composition

To describe the taxonomic composition of logged and unlogged plots, we used non-metric multidimensional scaling (NMDS) on a site-by-species abundance matrix; NMDS is a numerical ordination technique that maximizes the rank-order correlation between distance measures and distance in ordination space (Holland, 2008). The ‘stress’ value for an NMDS indicates how well the ordination summarizes the observed distances among samples (Holland, 2008), with values <0.2 generally considered a good fit. Species composition for each plot was characterized by its position in ordination space, represented by the scores on the first and second axes of the NMDS. These scores for logged and unlogged plots were then compared using a Student's unpaired *t*-test. We further compared species composition between logged and unlogged plots by using an indicator species analysis to identify species whose patterns of abundance were strongly associated with a particular logging status. The indicator species analysis is appropriate because it relates species abundance values from a set of sampled sites to the classification of the sites into independently predetermined groups (logged/unlogged; De Cáceres, 2013). The indicator species value is computed for each species independent of other species in the community and estimated as the product of a measure of its sensitivity and fidelity to each logging status category (Legendre and Legendre, 1998). Importance values for each species were estimated as the sum of percentage relative density and relative basal area in all logged plots pooled together and all unlogged plots pooled together. From the importance values, we determined the identity of the 10 species that make the most substantial contributions to the density and basal area of the logged and unlogged forest plots, respectively.

#### Structural composition

To compare structural differences between logged and unlogged plots, we calculated the following parameters.

- Plot stem density was calculated as number of stems per plot area (200 m × 10 m).
- Basal area was calculated as the sum of basal area for all trees in each plot, computed as π*r*^2^, where *r* is the radius of the tree, estimated as *r* = DBH/2.
- The coefficient of skewness (*g*_1_) was calculated to characterize the symmetry of tree size distributions in each plot (Bendel *et al.*, 1989; Wright *et al.*, 2003). When a plot is dominated by an abundance of small trees and a long tail of rare large trees, *g*_1_ is positive, whereas when the plot is dominated by an abundance of large trees and a long tail of rare small trees, *g*_1_ is negative (Wright *et al*., 2003). The value of *g*_1_ is computed as:
  (2) $$g_{1=}\frac{n\sum_{i}(x_{i}-\bar{x}{)}^{3}}{(n-1)(n-2)s^{3}}$$
  where *n* is the number of individuals in a plot, *x_i_* is the logarithm of the DBH for individual *i*, $\bar{x}$ is the mean of *x_i_*, and *s* is the standard deviation of *x_i_* (Bendel *et al.*, 1989; Wright *et al*., 2003).
- Above-ground biomass for each stem was calculated using the predictive allometric equations for estimating AGB in moist forest stands provided by Chave *et al*. (2005). For each tree in a plot, AGB was computed twice, once using an equation requiring (Eq. 3) and once not requiring (Eq. 4), height values.
  (3) $$\hat{{\text{AGB}}_{\mathrm{dh}}}=0.0509\times \text{WD}\times D^{2}\times H$$

(4) $$\begin{array}{ll} & \hat{{\text{AGB}}_{d}}=\text{WD}\times \exp\{-1.499+2.1481\times \ln(D)\\ & \;+0.207\times [\ln(D){]}^{2}-0.0281[\ln(D){]}^{3}\} \end{array}$$

where $\hat{{\text{AGB}}_{\mathrm{dh}}}$ is the estimated AGB based on a given stem's height and DBH, while $\hat{{\text{AGB}}_{d}}$ is the estimated AGB based on the stem's DBH. The WD (in kilograms per cubic metre) is the species average wood density, *D* is the stem DBH (in metres), and *H* (in metres) is the stem height. For each stem, *H* was estimated by interpolating from the stem's DBH, based on the KNP forest-wide regression equation predicting height as a function of DBH that is described above in the species functional trait section. The total estimated AGB in a plot is obtained by summing $\hat{\text{AGB}}$ for all stems within the plot. Both $\hat{\text{AGB}}$ estimates were natural logarithmically transformed for normality.

#### Functional composition

The functional composition of logged and unlogged plots was calculated for each trait (DBH_max_, *H*_max_ and WD) as the community-weighted mean (CWM) using the following different weightings: (i) relative basal area (BA); and (ii) relative abundance (ABD). The two weightings provide complementary perspectives on how the forests differ in community attributes by emphasizing contributions from different structural components of the forest, either individuals with large basal area or abundant small stems, respectively (Carreño-Rocabado *et al.*, 2012). Each set of CWM values for each trait was then compared between logged and unlogged plots using a Student's unpaired *t*-test.

Given that logging happened in a spatially structured way, logged plots occurred in more northerly latitudes than unlogged plots. This distribution results in the problem of simple pseudoreplication, in which ‘replicate’ plots within the two logging categories are not spatially interspersed across logging categories (Hurlbert, 1984). Consequently, spatial processes could influence forest structure and composition independent of logging effects (Lindenmayer and Laurance, 2012; Ramage *et al.*, 2013). To determine whether geographical space substantially explains some of the variation in forest structure and composition that is attributed to logging history, we included latitudinal co-ordinates of each vegetation plot as a covariate in an ANCOVA. This provides a more conservative test for the effect of logging status (environmental predictor) in the presence of latitude, a proxy for spatial predictors, reducing the risk of a type 1 error (Peres-Neto and Legendre, 2010). However, because latitude was never a significant predictor of any of the community attributes considered when included as a covariate in an ANCOVA (Supplementary Table A1), we report only the results for *t*-test comparisons of logged and unlogged plots.

All statistical tests were implemented in R version 2.15.1 (R Core Team, 2012), using the packages vegan (Oksanen *et al.*, 2015), FD (Laliberté and Legendre, 2010) and indicspecies (De Cáceres and Legendre, 2009).

## Results

### Light environment (prediction I)

**Figure 1:**
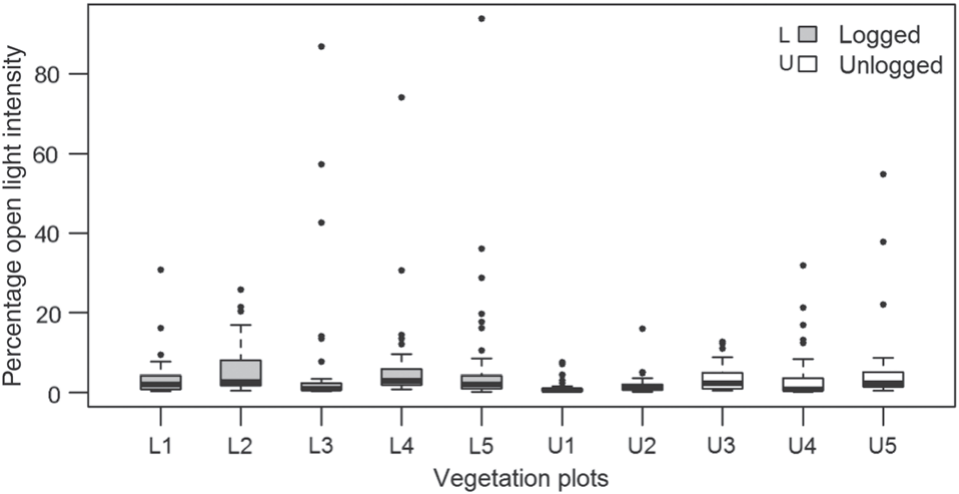
Percentage open light intensity for logged (L1–L5) and unlogged plots (U1–U5) in Kibale National Park (KNP), Uganda. Each vegetation plot has 42 light-intensity measurements. Each boxplot shows the median (black horizontal line), the upper quartile (space above black line), the lower quartile (space below black line), minimum (lower whisker) and maximum values (upper whisker) and extreme values or outliers (filled circles). Mean percentage open light intensity was significantly higher in logged plots (Student's unpaired *t* = 2.92, *P* < 0.05).

As predicted, logged forest plots had significantly higher light levels than unlogged plots (Fig. 1). However, light levels were not uniformly high within logged plots, because a large proportion of measured areas in logged plots was under shade (e.g. 77% of measured areas within logged plots had <5% open light intensity). Higher average light levels in logged plots resulted from light hotspots, i.e. extremely high values of percentage open light conditions recorded within logged plots (filled circles in Fig. 1).

### Structural composition (predictions II and III)

In total, 2358 stems ≥10 cm in DBH were inventoried in all 26 plots. Logged plots had significantly lower stem density, contrary to our expectation of higher stem density (prediction II); however, we did find support for lower total basal area and lower AGB (Table 1) in logged compared with unlogged forest.

**Table 1:** Mean (±1 SD) light availability, structural attributes and functional traits for logged (*n* = 9) and unlogged plots (*n* = 17) and results from a Student's unpaired *t-*test, including the test statistic (*t*) and degrees of freedom

Community attribute	Mean ± 1 SD for logged plots	Mean ± 1 SD for unlogged plots	*t*	d.f.
Structural composition				
Total basal area (cm^2^)	49 265 ± 22 021	89 210 ± 33 182	−3.67**	23
Stem density (stem number/m^2^)	0.03 ± 0.01	0.05 ± 0.01	−3.63**	16
AGB (with height; kg)	2262 ± 1139	5091 ± 2876	−3.56**	23
AGB (without height; kg)	930 ± 426	1840 ± 727	−4.02**	24
*g*_1_	0.58 ± 0.38	1.20 ± 0.53	−3.46**	22
Functional composition				
CWM WD_ABD_ (g/cm^3^)	0.57 ± 0.04	0.59 ± 0.02	−1.65	12
CWM WD_BA_ (g/cm^3^)	0.56 ± 0.03	0.59 ± 0.04	−2.17*	20
CWM *H*_max.ABD_ (m)	26.9 ± 3.8	27.9 ± 1.9	−0.72	10
CWM *H*_max.BA_ (m)	29.7 ± 5.3	32.2 ± 4.0	−1.25	13
CWM DBH_max.ABD_ (cm)	76.3 ± 8.7	76.7 ± 9.3	−0.12	17
CWM DBH_max.BA_ (cm)	95.6 ± 23.0	125.8 ± 53.1	−2.01	23

Abbreviations: ABD, abundance weighted; AGB, above-ground biomass; BA, basal area weighted; CWM, community-weighted mean; *H*_max_, maximum height; DBH_max_, maximum diameter; *g*_1_, coefficient of skewness; WD, wood density. Significant tests are indicated as follows: *P < 0.05 and **P < 0.01.

**Figure 2:**
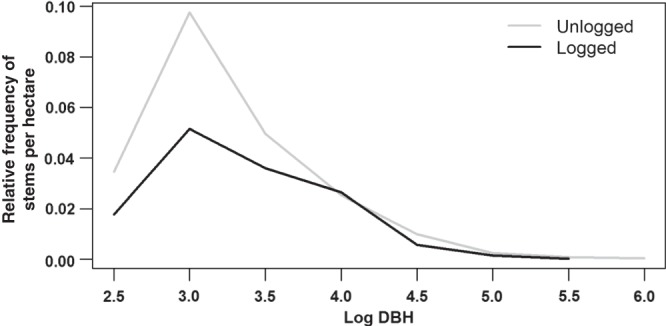
Diameter distributions for logged and unlogged plots in KNP. Lines show the relative frequencies of stem densities (*y*-axis) in each log-transformed diameter at breast height (DBH) size class (*x*-axis). Logged plots (*n* = 9) are represented by the black line and unlogged plots (*n* = 17) by the grey line.

Tree size distributions in logged plots (Fig. 2) had less positive *g*_1_ values than unlogged plots (Table 1), indicating that logged plots were dominated to a lesser extent by an abundance of small trees and had a shorter tail of rare large trees relative to unlogged plots. These different levels of asymmetry in tree-size distributions of logged and unlogged plots were in accordance with prediction III.

### Taxonomic composition (prediction IV)

**Figure 3:**
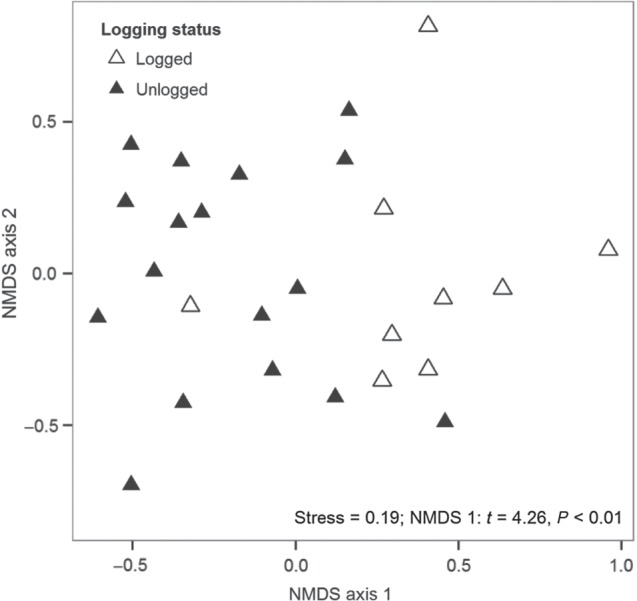
Ordination of taxonomic composition of logged (*n* = 9) and unlogged (*n* = 17) plots in KNP. The non-metric multidimensional scaling produced two axes (NMDS1 and NMDS2). Logged plots (open triangles) differed significantly from unlogged plots (filled triangles) in species composition along the first axis (*t* = 4. 26, *P* < 0.01) based on a Student's unpaired *t-*test, with most logged plots loading positively on axis 1.

A total of 86 tree species in 39 families were found across all 26 plots. The NMDS recovered two axes (Fig. 3), NMDS1 and NMDS2, with a stress value of 0.19, meaning it had a good fit. Logging status was best separated along the first axis of the NMDS (NMDS1; Fig. 3), with logged plots having higher scores than unlogged plots. Taxonomic turnover between logged and unlogged plots was within 2 units on NMDS1.

Only nine species were indicator species showing strong associations with logging status; four were strongly associated with unlogged and five with logged plots (Table 2). Logged and unlogged plots shared six of the 10 species with the highest importance values, but in different orders of importance (Table 3).

**Table 2:** Species (Family) with significant logging status associations, species' indicator values and the probability (*P*) of obtaining as great an indicator value as observed over 999 iterations

Indicator species	Logging status	Indicator value	*P*-value
*Vepris nobilis* (Rutaceae)	Unlogged	0.847	0.006
*Trilepisium madagascariense* (Moraceae)	Unlogged	0.825	0.016
*Leptonychia mildbraedii* (Malvaceae)	Unlogged	0.811	0.006
*Mimusops bagshawei* (Sapotaceae)	Unlogged	0.686	0.024
*Rothmannia urcelliformis* (Rubiaceae)	Logged	0.758	0.004
*Ehretia cymosa* (Boraginaceae)	Logged	0.722	0.011
*Euadenia eminens* (Capparaceae)	Logged	0.719	0.010
*Fagaropsis angolensis* (Rutaceae)	Logged	0.711	0.046
*Kigelia africana* (Bignogniaceae)	Logged	0.584	0.039

**Table 3:** Species (Family) with the 10 highest importance values in logged and unlogged plots, and their importance values^a^

Logged plots		Unlogged plots	
Important species	Importance value	Important species	Importance value
*Celtis durandii*^c^ (Cannabaceae)	25.0	*Celtis durandii* (Cannabaceae)	21.7
*Diospyros abyssinica* (Ebenaceae)	24.8	*Funtumia africana* (Apocynaceae)	18.5
*Funtumia africana* (Apocynaceae)	18.6	*Trilepisium madagascariense* (Moraceae)	18.0
*Markhamia lutea*^c^ (Bignoniaceae)	17.4	*Uvariopsis congensis*^c^ (Annonaceae)	14.5
*Celtis africana*^b^ (Cannabaceae)	11.6	*Diospyros abyssinica* (Ebenaceae)	13.4
*Premna angolensis* (Lamiaceae)	10.9	*Markhamia lutea* (Bignoniaceae)	10.9
*Strombosia scheffleri*^b^ (Olacaceae)	6.1	*Strombosia scheffleri* (Olacaceae)	9.6
*Millettia dura* (Leguminosae)	6.0	*Aningeria altissima*^b^ (Sapotaceae)	8.9
*Trilepisium madagascariense* (Moraceae)	5.6	*Vepris nobilis*^c^ (Rutaceae)	6.3
*Cordia africana* (Bignoniaceae)	5.0	*Pseudospondias microcarpa* (Anacardiaceae)	6.0

^a^The distribution of species importance values was similar for logged and unlogged plots; average (±1 SD) importance values were 2.38% (±4.42) for species in logged forest plots and 2.38% (±4.82) for species in unlogged forest plots. ^b^Species extracted for timber during the selective logging event (Struhsaker, 1997; Bonnell *et al.*, 2011). ^c^Species that may have suffered incidental damage during the selective logging event (Struhsaker, 1997).

### Functional composition (prediction IV)

Logged and unlogged plots did not differ significantly in functional composition, contrary to our prediction, because both plot types were similar in their community-weighted means for all three functional traits when weighted by abundance. The basal-area-weighted community mean WD was significantly lower in logged compared with unlogged plots, but the difference was small (Table 1).

## Discussion

Selective logging is a land-use practice that is increasing in extent and intensity and can alter the conservation value of tropical forests (Asner *et al.*, 2009). Previous investigations on the effects of selective logging on KNP's forests have focused mainly on demographic rates and structural attributes of the forest (Chapman and Chapman, 1997, 2004; Bonnell *et al.*, 2011). Here, we advance the investigation of the effects of selective logging on KNP's forests by focusing on the understory light availability and the structural, taxonomic and functional attributes of the forest 45 years after the selective logging event occurred. We found that on average the light levels were higher in logged forest, but with lower stem density, smaller total basal area and AGB and a dissimilar species composition in comparison to unlogged forest. Nevertheless, differences in the taxonomic and structural composition of logged forests were not paralleled by differences in community-weighted average trait values; logged plots were functionally analogous to unlogged plots except when community mean WD was basal area weighted.

### Logged forest: higher light environment

Some tropical forests achieve canopy closure after a few decades (Asner *et al.*, 2004). For example, Nicotra *et al.* (1999) reported no differences in average light levels between logged and unlogged forest in Costa Rica 15–20 years after selective logging. However, this pattern appears to be less true for African forests. Even after 45 years, logged plots on average had almost double the light intensity of unlogged plots (Fig. 1), probably due to canopy gaps failing to close.

The distribution of light levels within each plot (Fig. 1) suggests a preponderance of shaded light conditions in both logged and unlogged plots, with a few extreme light hotspots driving average light levels higher in logged forest. The primary source of shade in unlogged plots at 2 m is the closely connected tree canopy, but in logged plots dense herbaceous or shrubby growth overtakes canopy gaps and is a large contributor to the shade at 2 m (Supplementary Fig. A1 is a picture of a quantum sensor in shade from shrubby growth). Given that these shrubs (often *Acanthus pubescens*) are both clonal and browse tolerant, they are more successful than trees in large canopy gaps, such as those created during the logging event and now maintained by large mammal herbivory in KNP (Struhsaker, 1997; Paul *et al.*, 2004; Lawes and Chapman, 2006; Royo and Carson, 2006; Young and Peffer, 2010).

### Logged forest: distinct structural composition

In typical disturbed forests, most stems are in small size classes at high stem densities, with few large trees (Denslow, 1995). In KNP, distributions for both logged and unlogged forest approximated the ‘reverse J-shape’ or negative exponential distribution (Richards, 1996), which implies an uneven age structure, with an abundance of small relative to large-sized tree classes (Fig. 2). However, logged plots had less positively skewed distributions than unlogged plots. The less positive skew derived from both an absence of large and a low proportion of small-sized stems in logged forest (Fig. 2). This result is consistent with previous reports from KNP that logged plots experienced reduced recruitment but similar mortality rates of adult trees (≥10 cm DBH) to unlogged plots in the first 31 years after logging (Bonnell *et al*., 2011).

Logged plots in KNP also had fewer total stems per unit area and on average held 44% less AGB than unlogged plots (Table 1). The lower AGB is likely to have resulted from both the low stem density and the scarcity of larger trees in logged plots, because large trees make disproportionate contributions to AGB and carbon storage (Lindner and Sattler, 2012).

The absence of large trees in logged plots implies that insufficient time has passed for recovery of biomass, especially through the growth of remnant trees into large size classes. The low number of small trees in logged plots suggests reduced recruitment, which may have resulted from a number of factors. Unfavourable environmental conditions associated with tree damage and canopy loss may have limited recruitment (Chazdon, 2003; Hall *et al.*, 2003). Given that all but a few tree species in KNP perform poorly in large-gap conditions (Chapman *et al.*, 1999), sudden crown exposure may have caused physiological stress, limiting tree regeneration (Hall *et al*., 2003). Moreover, tree growth and recruitment may have been slowed by early competition from the rapid establishment of dense herbaceous and shrubby vegetation (Donato *et al.*, 2012) and increased elephant activity. Elephants are known to forage extensively on these shrubs (Paul *et al.*, 2004; Lawes and Chapman, 2006; Duclos *et al.*, 2013; Omeja *et al.*, 2014). Based on the measured structural attributes and continued dominance of herb and shrub vegetation, our study supports previous findings that logged forest in KNP is in an arrested state of succession (Chapman and Chapman, 1997, 2004; Bonnell *et al.*, 2011).

The persistence over several decades of the effect of selective logging on forest structure is not unique to KNP among African forests. For example, Plumptre (1996) reported that 50 years after, logged compartments in Budongo Forest Reserve, East Africa, had not recovered to pre-logging levels in measures of forest structure, including mean basal area and crown height. Likewise, Hall *et al*. (2003) report markedly lower basal area and significantly lower stem densities 18 years after logging in a Central African forest. However, in contrast to KNP, another Central African forest showed rapid recovery in AGB within 20 years after logging (Gourlet-Fleury *et al.*, 2013). Reports seem to vary across studies depending on the measure of forest structure and also due to differences in site-specific factors, such as selective logging intensity and the presence of large mammal herbivory.

### Logged forest: divergent taxonomic but analogous functional composition

The taxonomic composition of plots differed depending on the logging history (Fig. 3), although the nature of these differences was surprising. We found considerable overlap in important species between logged and unlogged plots; however, the order of importance for these overlapping species differed (Table 3). Also, the five species that were closely associated with logged plots (Table 2) were occasional to rare (<20 stems in total), occurring almost exclusively in logged plots. These results suggest that while the selectively logged environment may have favoured the establishment of a few rare disturbance-adapted species, most of the taxonomic differences associated with logging result from changes in relative abundances of species.

**Figure 4:**
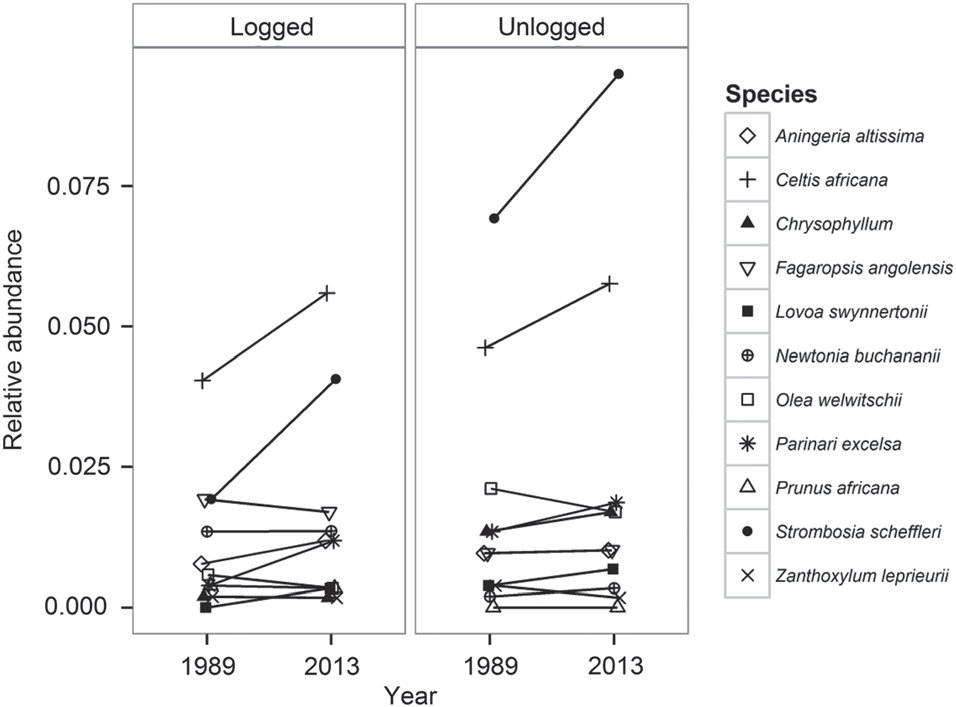
Trends in relative abundances of 11 tree species commercially logged in KNP, based on their relative abundances in 1989 and 2013 in logged and unlogged plots. Each species is represented by a different symbol, while lines connect symbols to show trends between relative abundance in 1989 and 2013 for each species.

While we did not find strong taxonomic differences between logged and unlogged forest, these differences may be apparent when contrasting the relative abundances of tree species that were commercially exploited during the logging event. In examining these patterns, however, no obvious differences between 1989 and 2013 can be seen for 11 species commercially harvested in Kibale (Fig. 4). While two species (*Celtis africana* and *Strombosia scheffleri*) increased in relative abundance, only *S. scheffleri* had higher relative abundance in unlogged plots. The other commercially exploited species, including the iconic *Parinari excelsa*, maintained a consistent trend of low relative abundances (<0.025) across logged and unlogged plots (Fig. 4). It is unclear whether the lack of recovery for the majority of these commercial species is related to regeneration or recruitment, particularly because the two species with increasing abundances represent contrasting ecological strategies. *Celtis africana* is a disturbance-tolerant small-statured generalist with very small seeds and low leaf toughness, while *S. scheffleri* is a shade-loving understory species with larger seeds and high leaf toughness (Chapman *et al.*, 2008; Neuschulz *et al.*, 2013). Moreover, Chapman and Chapman (2004) reported forest-wide fruiting declines for *P. excelsa* and *Aningeria altissima* (another commercially exploited species showing poor recovery), which they linked to climate change. Taken together, these results suggest that observed taxonomic patterns are generated by multiple perturbations, including forest-wide disturbances unrelated to selective logging.

Differences in functional composition between sites with varying logging histories have been found in other studies (Baraloto *et al.*, 2012; Carreño-Rocabado *et al.*, 2012). In contrast, we do not see a difference in CWM values for WD, *H*_max_ and DBH_max_ between logged and unlogged plots, except for when WD was basal area weighted, although this difference was modest (0.03 g/cm^3^; Table 1), suggesting that selective logging may have favoured the increased growth of species with slightly lower WD.

There are several plausible explanations for this pattern of functionally analogous communities in logged and unlogged forests. First, KNP forest has a small species pool of 86 tree species within 5.2 ha; this is consistent with the general trend of less diversity in African forests relative to tropical forests in other regions (Kreft and Jetz, 2007). A poor species pool can limit trait variation within communities (Mayfield *et al.*, 2010). Moreover, KNP's poor species pool lacks aggressive colonizing tree species (Chapman *et al*., 1999), which tend to have lower WD and smaller *H*_max_. When comparing KNP with other tropical locations, KNP appears to resemble the species pool for Africa more generally in having a greater incidence of intermediate WD species and an absence of species with extreme WD values (Supplementary Fig. A2). Even naturally occurring tree-fall gaps in KNP are characterized by a similar community composition to the forest interior (Zanne and Chapman, 2005), rather than being dominated by pioneer species. Furthermore, Chapman and Chapman (2004) observed a decline in the abundance of the few pioneer species in logged forests in KNP, e.g. *Trema orientalis*. In our study, the five species that showed tight associations with the logged forest were small sized and soft wooded, but their modest additions to relative stem density and basal area meant that they had little influence on average community-weighted values. Second, both logged and unlogged forests may be undergoing change triggered by disturbance events independent of the logging event 45 years ago. Such events have recently been reported for KNP, including changing climates (i.e. longer drought events since the mid-1990s (Hartter *et al.*, 2012) and increased elephant abundance and activity (Omeja *et al.*, 2014).

### Caveat

As is common in logging-impact studies, in this study the logging history is confounded with geographical space; the logging treatment was implemented as a forestry practice and not for scientific research (Lindenmayer and Laurance, 2012; Ramage *et al.*, 2013). Previous studies of KNP have assumed that compartments within which vegetation plots are located were structurally similar before logging, based on historical ground surveys that predate the logging event (Kingston, 1967; Chapman and Chapman, 1997; Bonnell *et al.*, 2011). Here, in addition to the assumption of pre-logging structural similarity of plots, we applied a simple approach to account for pseudoreplication by including latitudinal geographical co-ordinates for each observation as a covariate in ANCOVA tests. This covariate was not significant for any measure of forest composition and structure (Supplementary Table A1), increasing our confidence that the results we found were due to logging history. However, we are unable to test more explicitly for the effects of spatial processes, and our results should be interpreted with this caveat in mind.

### Conclusion

Selective logging is a land-use practice that is becoming increasingly widespread in the tropics, although the extent to which it impacts the taxonomic, structural and functional composition of forests remains unclear. If we assume that unlogged forest is less disturbed than logged forest, from our work, we conclude that 45 years is not enough time for selectively logged forests in KNP to recover in species composition and structural complexity, but enough time for community-weighted traits to resemble unlogged forest. This functional similarity can be understood in the context of the ecology of Africa generally and KNP more specifically, including a lack of aggressive colonizing trees, a relatively small species pool, concentrated elephant activities and potential background changes in both forest types unrelated to the selective logging event. Despite the functional similarity in community average WD and adult stature, the dearth of large trees and small stem density reduced the capacity of the logged forest for carbon storage, as evidenced by significantly less AGB in logged plots. It is likely that the effect of selective logging that has persisted in KNP forest results in part from poor tree recruitment and high mortality of existing trees in the logged plots soon after the logging event.

Consequently, from a conservation standpoint, our results suggest that caution should be taken when considering the conservation value of selectively logged forests (Putz *et al.*, 2012; Edwards and Laurance, 2013; Michalski and Peres, 2013; Kormos and Zimmerman, 2014). Certainly, given its similar functional trait and not overly distinct taxonomic composition compared with unlogged forest, logged forest in KNP holds greater conservation value than surrounding areas subjected to farming and plantation forestry (Okiror *et al.*, 2012). Nevertheless, some argue that for selectively logged forests to have high conservation value they must display rapid recovery following the logging event (Michalski and Peres, 2013). We show in this study that a tropical forest may remain with the imprint of logging for many decades. Furthermore, persistent effects of selective logging have exerted cascading effects on other trophic levels, particularly affecting the population dynamics of primates (Chapman *et al.*, 2010b; Bonnell *et al.*, 2011) and movements of elephants (Omeja *et al.*, 2014). Interestingly, logging extraction levels in KNP ranged from 14 to 17 m^3^/ha (Struhsaker, 1997), comparably less than the 32.5 m^3^/ha reported by Blanc *et al.* (2009) for a selectively logged forest in French Guiana that rapidly recovered AGB within 40 years. Whether this difference in recovery represents a general contrast between the vulnerabilities to anthropogenic disturbances of mid-elevation and lowland forests or is more due to site-specific differences (e.g. concentrated elephant activities in KNP) remains to be seen. Until we have better answers, strategies for sustainably managing and conserving tropical forests should be informed by local forest dynamics and vulnerabilities to disturbance, rather than blanket ‘one size fits all’ conclusions on the conservation value of logged forests (Corlett and Primack, 2006).

## Funding

This work was supported by the Whitney Harris World Ecology Center [to O.L.O.]; the Webster Groves Nature Study Society [to O.L.O.]; IDEA WILD [to O.L.O.]; NSERC [to C.A.C.]; FQRNT [to C.A.C.]; and the National Institutes of Health [grant TW009237 to C.A.C.] as part of the joint NIH-NSF Ecology of Infectious Disease programme and the UK Economic and Social Research Council.

## Supplementary Material

Supplementary Data
