# Supplementary material for: Selective logging: does the imprint remain on tree structure and composition after 45 years?
Source: Conserv Physiol. 2015 Mar 24;3(1):cov012. doi: 10.1093/conphys/cov012 (PMC4778436; doi:10.1093/conphys/cov012)
Supplement: Supplementary Data [file supp_3_1_cov012__index.html]

Supplementary Data 

# Selective logging: does the imprint remain on tree structure and composition after 45 years?

## Supplementary Data

Supplementary Data

**Files in this Data Supplement:**

- Supplementary Data - Docx file
