# Supplementary material for: Selective logging: does the imprint remain on tree structure and composition after 45 years?
Source: Conserv Physiol. 2015 Mar 24;3(1):cov012. doi: 10.1093/conphys/cov012 (PMC4778436; doi:10.1093/conphys/cov012)
Supplement: Supplementary Data [file supp_cov012_cov012supp.docx]

**Supplementary data**

Table A1. Effect of logging status on all community attributes in the presence of a spatial covariate (latitude) with the *F* statistic for main effects of logging status and latitude reported for Analysis of Covariance (ANCOVA) tests run for each community attribute. Significant tests are in bold (** *P* < 0.01, * *P* < 0.05). Acronyms are defined as follows: CWM = community weighted mean, WD = wood density, H_MAX_ = maximum height, DBH_MAX_ = maximum diameter, ABD = abundance weighted, AGB = aboveground biomass BA = basal area weighted, and g*_1_* = coefficient of skewness.

| **Community attribute** | **Logging status** | **Latitude** |
| --- | --- | --- |
|  | ***F*** | ***F*** |
| **Structural composition** |  |  |
| Light intensity (% open light intensity) | **11.333*** | 3.655 |
| Total basal area (cm^2^) | **10.261**** | 0.492 |
| Stem density (#/m^2^) | **13.21**** | 0.420 |
| Relative gap phase | 0.034 | 0.074 |
| *g_1_* | **9.842**** | 1.294 |
| AGB (with height; kg) | **14.142**** | 0.131 |
| AGB (without height; kg) | **17.136***** | 0.019 |
| **Species composition** |  |  |
| NMDS1 | **19.028***** | 0.522 |
| NMDS2 | 0.000 | 1.234 |
| **Functional composition** |  |  |
| CWM WD_ABD_ (g/cm^3^) | 3.44 | 0.020 |
| CWM WD_BA_ (g/cm^3^) | 3.906 | 0.108 |
| CWM H_MAX.ABD_ (m) | 0.724 | 0.004 |
| CWM H_MAX.BA_ (m) | 1.823 | 0.475 |
| CWM DBH_MAX.ABD_ (cm) | 0.015 | 2.746 |
| CWM DBH_MAX.BA_ (cm) | 2.523 | 0.247 |


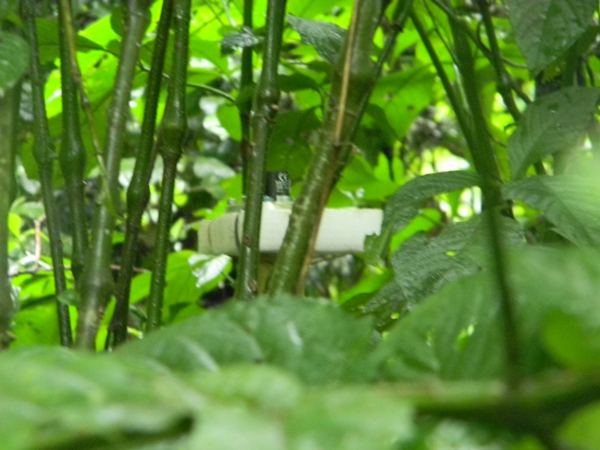


Fig A1. Photo of a quantum sensor at 2 m above ground level underneath shade exerted by dense herbaceous growth in logged plot in Kibale National Park, Uganda. Source: Oyomoare Osazuwa-Peters


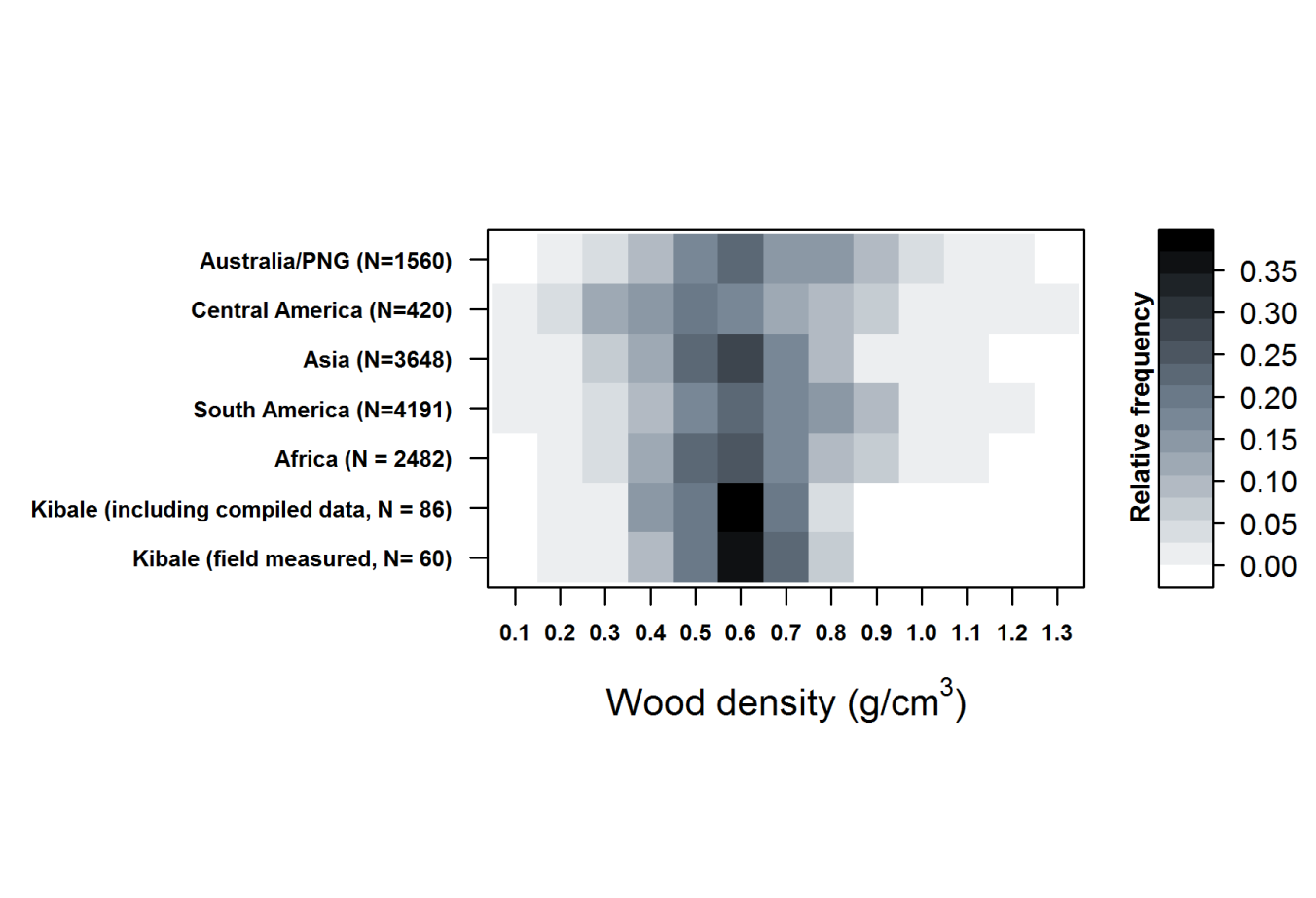


Fig. A2. Map of relative frequencies of wood density values of species in KNP forest for only field measured data (*N* = 60), and with field measured and compiled data from a subset of the Global Wood Density Database for African region included (*N*=86), as well as for the Global Wood Density Database subset for tropical Africa (*N* = 2482), tropical South America (*N* = 4191), tropical Asia (*N=*3648), tropical Central America (*N*=420), and tropical Australia/Papau New guinea (PNG) (*N*=1560). Color scheme ranges from zero frequency (white) to the highest frequency (black).
